# One-Step Preparation of Highly Stable Copper–Zinc Ferrite Nanoparticles in Water Suitable for MRI Thermometry

**DOI:** 10.1021/acs.chemmater.2c00079

**Published:** 2022-04-20

**Authors:** Dorota Lachowicz, John Stroud, Janusz H. Hankiewicz, River Gassen, Angelika Kmita, Joanna Stepień, Zbigniew Celinski, Marcin Sikora, Jan Zukrowski, Marta Gajewska, Marek Przybylski

**Affiliations:** † Academic Centre for Materials and Nanotechnology, AGH University of Science and Technology, 30-059 Krakow, Poland; ‡ Center for the Biofrontiers Institute, University of Colorado Colorado Springs, 1420 Austin Bluffs Pkway, Colorado Springs, Colorado 80918, United States; § Faculty of Physics and Applied Computer Science, AGH University of Science and Technology, 30-059 Krakow, Poland

## Abstract

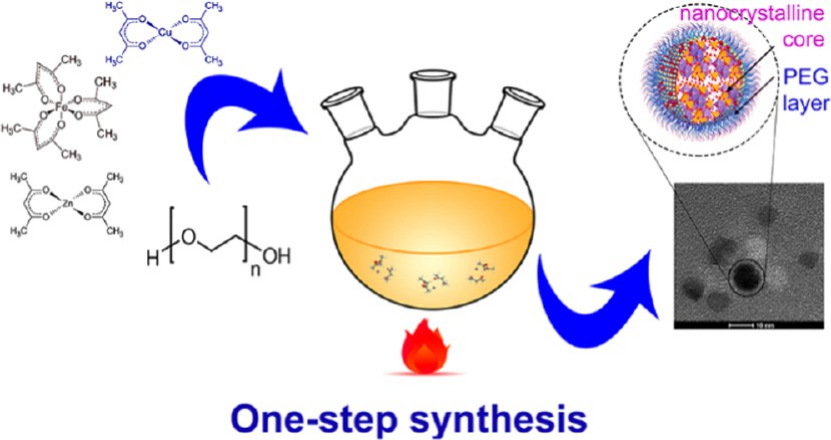

Superparamagnetic ferrite nanoparticles coated with a polymer layer are widely used for biomedical applications. The objective of this work is to design nanoparticles as a magnetic resonance imaging (MRI) temperature-sensitive contrast agent. Copper–zinc ferrite nanoparticles coated with a poly(ethylene glycol) (PEG) layer are synthesized using a one-step thermal decomposition method in a polymer matrix. The resulting nanoparticles are stable in water and biocompatible. Using Mössbauer spectroscopy and magnetometry, it was determined that the grown nanoparticles exhibit superparamagnetic properties. Embedding these particles into an agarose gel resulted in significant modification of water proton relaxation times *T*_1_, *T*_2_, and *T*_2_* determined by nuclear magnetic resonance measurements. The results of the spin-echo *T*_2_-weighted MR images of an aqueous phantom with embedded Cu_0.08_Zn_0.54_Fe_2.38_O_4_ nanoparticles in the presence of a strong temperature gradient show a strong correlation between the temperature and the image intensity. The presented results support the hypothesis that CuZn ferrite nanoparticles can be used as a contrast agent for MRI thermometry.

## 1 Introduction

Part of the evolution from anatomical to functional magnetic resonance imaging (MRI) is the development of the method for a noninvasive temperature mapping to help in the diagnosis of some pathological changes that can be correlated with temperature increases^1^ or to use during MRI-guided thermal ablation procedures.^2^ Currently, the proton resonance frequency shift (PRF) method is the gold standard used to measure temperature by phase changes in MR images. However, the presence of adipose tissue, magnetic field drift, or tissue movement and their heterogeneity easily inhibits it. The use of nanoparticles (NPs) for magnetic resonance imaging thermometry (tMRI) measurements could overcome these limitations, but if nanoparticles are to be used as a contrast for tMRI, they must have appropriate magnetic properties. The presented research describes the development of a novel temperature-sensitive contrast agent suitable for magnetic resonance imaging thermometry (tMRI).^3^

In the past, we demonstrated^4−7^ that the magnetic particles of micron size or clusters of nanoparticles 200 nm in size can be used to determine the absolute temperature from the intensity of *T*_2_*-weighted gradient-echo MRI images.^8,9^ For clinical applications, however, the use of such large particles is not possible. For this reason, we have focused our research on the preparation of magnetic ferrite nanoparticles whose properties can be optimized for use as an intravenous contrast agent in MRI-guided thermal treatment procedures.

In previous studies, we successfully used gadolinium^10^ particles to measure temperatures with an accuracy on the order of 1 °C as a result of induced brightness changes in *T*_2_*-weighted MR images.^4^ The temperature contrast in MRI can be achieved by fabricating nanoparticles with Curie temperature, *T*_C_, near the temperature region of interest (ROI). In previous studies, we successfully used gadolinium particles (*T*_C_ = 19 °C^10^) to measure temperatures with an accuracy on the order of 1 °C as a result of induced brightness changes in *T*_2_*-weighted MR images.^4^ Because of a similar radius, Gd^+3^ is known to be toxic due to blocking the calcium channel.^11^ Gadolinium ions contained in chelates are well-established and safe agents used to contrast anatomical MR images. Although an increased level of Gd ions was recently linked with nephrogenic systemic fibrosis,^12,13^ the risk is rather low.^14^ Micrometer size metallic gadolinium particles can be safe by coating them with inert metals such as gold. However, sequestrating nanosize metallic gadolinium is difficult and may not prevent the leaching of Gd^+3^ ions. This is why ferrite compounds, known for their good biocompatibility, have been subsequently investigated as alternative tMRI contrast agents,^7,8^ and the preparation of ferrites in the form of nanoparticles is essential for this project.

Ferrite nanoparticles exhibit superparamagnetic properties, but most of them are prone to poor chemical stability. As a result, there is a need to modify the surface or add other elements.^15−18^ Additionally, ferrite nanoparticles have a high surface-to-volume ratio and tend to aggregate. Therefore, it is necessary to develop a strategy for their *in vivo* applications to prevent aggregation.

The main goal of our work is to obtain (in a one-step, low-cost process) stable in water, biocompatible nanoparticles that can be used as a tMRI contrast agent. The use of nanoparticles as an *in vivo* contrast also requires the elimination of toxic substances from the production process and the stabilization of the surface of the particles with a polymer coating that will prevent aggregation,^19,20^ opsonization,^21^ and interaction with the elements of the immune system. The formation of nanoparticle aggregates influences their biodistribution *in vivo*. Literature reports indicate that smaller particles longer remain in the bloodstream.^22^

PEGylation of nanoparticles can reduce the formation of *in vivo* aggregates and prolong the circulation time in the bloodstream to the time necessary for the completion of MRI-guided thermal ablation. Specifically, laser ablation requires three to five repetitions, 2 min each, depending on the number of tumors and tumor size.^23^

For nanoparticles to be employed as a contrast agent in humans, they must be dispersible in water.^24^ Currently, two methods are mainly used for the synthesis of hydrophilic magnetic nanoparticles: (1) coprecipitation reactions in the presence of surfactants^25−28^ and (2) a two-step process consisting of the production of nanoparticles by the thermal decomposition of organometallic compounds in an organic solvent, followed by surface modification of the particle.^29−32^ The most commonly used coatings for magnetic nanoparticles are proteins,^33,34^ natural polysaccharides,^22,25^ synthetic polymers such as poly(vinyl alcohol),^33,34^ poly(vinylpyrrolidone),^35−38^ poly(ethylene glycol) (PEG),^39,40^ poly(lactic-*co*-glycolic) acid,^41,42^ and also precious metals (gold or silver).^43−45^ The multistep techniques typically include methods for the thermal decomposition of organometallic precursors (e.g., Fe(acac)_3_) in a high boiling solvent in the presence of stabilizing surfactants such as oleylamine^46^ or/and oleic acid. Currently, many groups are developing thermal decomposition methods in mixtures of surface modifying substances for the direct synthesis of water-soluble magnetic nanoparticles;^38,46−50^ however, such process still needs to be improved.

This work presents the synthesis of copper–zinc ferrite nanoparticles coated with PEG with different copper concentrations. We investigated the influence of increasing the content of copper ions in the reaction mixture on the structure of the polymer shell of the obtained nanoparticles using X-ray photoelectron spectroscopy (XPS). The XPS analysis allowed us to determine the differences in the structure of the polymer layer stabilizing the synthesized nanoparticles and their chemical composition. Finally, we also determined the surface charge, size, and morphology of the nanoparticles as well as their magnetic properties using a variety of structural and magnetic techniques. To determine the cytotoxicity *in vitro*, we exposed several selected cell lines to the produced nanoparticles. We have shown here that by embedding PEG-coated nanoparticles in an agar gel phantom, we could measure its temperature due to temperature-dependent changes in the brightness of the *T*_2_ weighted spin-echo MR images.

## 2 Experimental Section

### 2.1 Materials

Copper(II) acetylacetonate (≥99.9% trace metals basis), zinc(II) acetylacetonate, iron(III) acetylacetonate (puriss. P.a., 99.9%), and poly(ethylene glycol) (1000 Da, BioUltra) were used as received (all from Sigma-Aldrich, St. Louis, MO). Acetone (puriss. P.a) and diethyl ether (puriss. P.a.) were purchased from POCH/Avantor (Gliwice, Poland). Deionized water was used for all solutions.

### 2.2 Microscopy Methods

The transmission electron microscopy (TEM) images were acquired with a Tecnai TF 20 X-TWIN microscope (FEI, Hillsboro, OR). The TEM pictures were analyzed using Image-J software. The AFM images of particles were acquired with a Dimension Icon XR atomic force microscope (Bruker, Santa Barbara, CA) working in the air in the PeakForce Tapping mode using standard silicon support cantilevers with a nominal spring constant of 0.4 N/m (tip radius < 8 nm).

### 2.3 Inductively Coupled Plasma Optical Emission Spectrometry (ICP-OES)

The chemical composition of nanoparticles was studied with inductively coupled plasma optical emission spectroscopy (ICP-OES; iCAP7400 Plus instrument, Thermo Scientific, Bremen, Germany). All samples were dissolved in a concentrated Suprapur nitric acid.

### 2.4 X-ray Photoelectron Spectroscopy (XPS)

The XPS spectra were measured using a PHI 5000 VersaProbe II spectrometer with an Al K_α_ radiation source, *E* = 1486.6 eV (ULVAC-PHI, Chigasaki, Japan). The working pressure in the analytical chamber was less than 3 × 10^–7^ Pa. The high-resolution spectra were measured at the analyzer pass energy set to 49.95 eV. A dual-beam charge neutralizer was used to compensate for charging. All binding energies were corrected to the C–C line at 284.8 eV. The spectrum background was subtracted by the Shirley method. The data analysis was performed using PHI MultiPak software.

### 2.5 Fourier Transform Infrared Spectroscopy (FTIR)

The FTIR spectra were recorded with a spectrometer FTIR Tensor II (Bruker Optik GmbH, Ettlingen, Germany).

### 2.6 Dynamic Light Scattering (DLS)

The hydrodynamic diameter and the ζ-potential of the resulting particles in aqueous solutions were measured using the dynamic light scattering (DLS) method (Malvern Nano ZS, Malvern Instrument Ltd., Worcestershire, U.K.).

### 2.7 Thermogravimetric Analysis (TGA)

The thermal analysis was carried out using a thermogravimetric analyzer Q600 (TA Instruments, New Castle, DE). The analysis was conducted from room temperature (RT) up to 900 °C (heating rate of 10 °C/min) under a continuous flow of inert gas (100 mL/min). The data were used to determine the percentage of the organic compound in the obtained nanoparticles.^47,48^

### 2.8 Mössbauer Spectroscopy

The ^57^Fe Mössbauer spectra were measured in the transmission mode at room temperature (RT) and 80 K using a ^57^Co source in an Rh matrix. An electromechanical type Mössbauer spectrometer was operating in a constant acceleration mode. The 14.4 keV γ-rays were detected with a proportional counter (MS-4 spectrometer, Renon, Krakow, Poland). The velocity scale was calibrated at room temperature with a metallic iron foil. The low-temperature spectra were analyzed using the least-squares fitting procedure with several magnetically split spectrum components corresponding to different iron positions.

### 2.9 X-ray Absorption Spectroscopy (XAS)

The X-ray absorption spectroscopy (XAS) measurements were performed at the PEEM/XAS beamline of the Solaris National Synchrotron Radiation Centre, Krakow, Poland.^51^ The XAS bending magnet-based beamline provides a soft X-ray energy range and is equipped with a plane grating monochromator of a resolving power of *E*/Δ*E* > 4000. The XAS spectra were recorded at Zn, Cu, and Fe L2,3 edges in the total electron yield (TEY) detection mode (for Zn and Cu) and fluorescence yield (FY) detection mode (for Fe and Cu) at room temperature.

### 2.10 Magnetometry

The magnetization measurements as a function of temperature were conducted using a superconducting quantum interference device (SQUID) magnetometer in the temperature range of 4–350 K (Magnetic Property Measurement System by Quantum Design, San Diego, CA). At 4 mT, the measurements were carried out using a zero-field cooled (ZFC) and a field cooled (FC) protocols, having the sample temperature first lowered to 4 K in a field of 3.0 T. The mass magnetization was calculated using the corrected mass value from thermogravimetric measurements that show that the ferrite constitutes only 56.4% of the total sample mass.

### 2.11 Nuclear Magnetic Resonance Spectroscopy (NMR)

Nanoparticles with the formula Cu_0.08_Zn_0.54_Fe_2.38_O_4_ (S1 system) were chosen for subsequent NMR and MRI studies. The particles were embedded in 0.85 mM concentration in a 1% deionized water agar (Fisher Bioreagents, Ottawa, Canada) gel matrix to form NMR samples in standard 5 mm glass tubes (Wilmad-LabGlass, Vineland, NJ). The same doped agar was used to make an MRI phantom, as described below.

A pulse NMR spectrometer operating at 3.0 T (128,015.2 kHz) was used to determine the temperature dependence of the NMR line width for the *T*_2_* calculations. The nuclear spin–lattice (*T*_1_) and spin–spin (*T*_2_) relaxation times of water protons were measured in pure 1% deionized water agar gel and 1% agar gel with embedded copper–zinc ferrite nanoparticles. The NMR system used a Redstone console (Tecmag, Houston, TX) and a standard bore 54 mm superconducting magnet (Oxford Instruments, Abingdon, U.K.).^52^ The temperature-dependent measurements were conducted in the range from 5 to 50 °C with 5 °C increments. At each point, the sample’s temperature was stabilized with an accuracy of ±0.25 °C using the flow of heated nitrogen gas and a software-based proportional-integral-differential temperature controller with feedback from two platinum temperature sensors located near the sample. For the line width measurements, a one-pulse sequence was used with the following parameters: 90° pulse = 14.4 μs and repetition time (TR) = 3.5 s. The *T*_1_ measurements in agar gel with particles were conducted using the inversion-recovery (IR) method with the following pulse parameters: inversion 180° pulse = 28.8 μs, sampling 90° pulse 14.4 μs, with the inversion time (TI) array consisting of 20 delays from 9 ms to 10 s, and TR = 3.5 s. For the *T*_1_ measurements in pure agar gel, the same IR sequence was used with a longer TR of 20 s and a wider TI array covering the range from 40 ms to 40 s. The *T*_2_ relaxation time was measured with the Carr Purcell Meiboom Gill (CPMG) pulse sequence with the following parameters: 90° excitation pulse = 14.4 μs and 180° refocusing pulse = 28.8 μs. For the CPMG experiments in pure agar, 20 delays covered the range from 25 to 493 ms, with the TR = 20 s. During the *T*_2_ measurements of agar with embedded nanoparticles, the CPMG delays ranged from 1 to 39 ms, with the TR= 3.5 s.

### 2.12 Magnetic Resonance Imaging (MRI)

**Figure 1:**
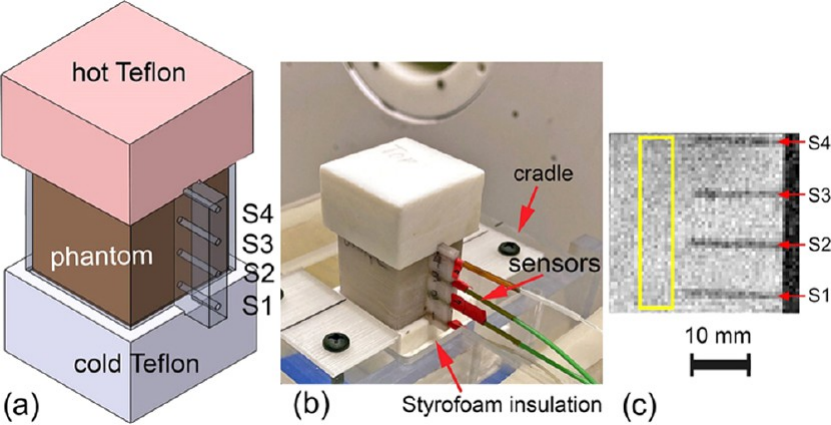
MR imaging in the presence of a strong temperature gradient. (a) Temperature cell with a gradient produced in the phantom by cold and hot blocks of Teflon. (b) Photograph of the assembled setup on an MRI-compatible cradle before insertion into the magnet. Note: Styrofoam insulation separating cold Teflon block from the cradle. (c) Sagittal cross-sectional MRI scout showing the positions of the temperature sensors in the cell in relation to the axial slice location marked as a yellow rectangle. The slice was subsequently used for the analysis of image intensity as a function of temperature.

The MRI experiments in the presence of a temperature gradient were conducted using a custom-designed three-dimensional (3D)-printed thin wall cell made of poly(lactic acid) (PLA). The phantom cell, shown in Figure 1a, was filled with 22.7 mL of agar gel doped with the 0.85 mM concentration of Cu_0.08_Zn_0.54_Fe_2.38_O_4_ (S1) nanoparticles. The cell was sandwiched between two Teflon blocks (36 × 36 mm^2^ and 20 mm high) to achieve the temperature gradient (Figure 1a,b). Before assembly, the bottom block was cooled to −30 °C, while the top block was heated to +90 °C. At the same time, the cell containing the phantom with the nanoparticle-doped agar gel was cooled to 10 °C. The top of the cell with exposed agar gel was covered with a thin layer of water to improve the thermal contact with the hot Teflon block. Four subminiature birefringent optical temperature sensors were inserted in the cell for the temperature measurements (±0.3 °C accuracy) as marked by OS1 through OS4 (AccuSens, Opsens, Quebec, Canada). The sensors are visible on the scout image in Figure 1c as four dark horizontal lines. The sensors’ locations relative to the cell bottom are 2, 10, 18, and 26 mm. The temperature recording data and analysis of the temperature distribution are presented in the Results and Discussion section above.

The MRI experiments were carried out in an Agilent preclinical scanner with a 3.0 T, 30 mm bore magnet (Agilent, Santa Clara, CA).^53^ After assembly, the temperature cell was placed in the magnet’s isocenter on a dedicated cradle with Styrofoam insulation to avoid direct thermal contact with the MRI bird-cage resonator. Three consecutive spin-echo images were acquired using the following parameters: slice orientation—axial, field of view (FOV) 35 × 35 mm^2^, matrix 64 × 64 (in-plane resolution—0.55 mm/pixel), slice thickness—6 mm, repetition time (TR)—4 s, echo time (TE)—20 ms, and image acquisition time—4 min 16 s. The location of the axial slice is shown on the sagittal scout image in Figure 13c as a yellow rectangle.

### 2.13 Preparation of Nanoparticles

**Figure 2:**
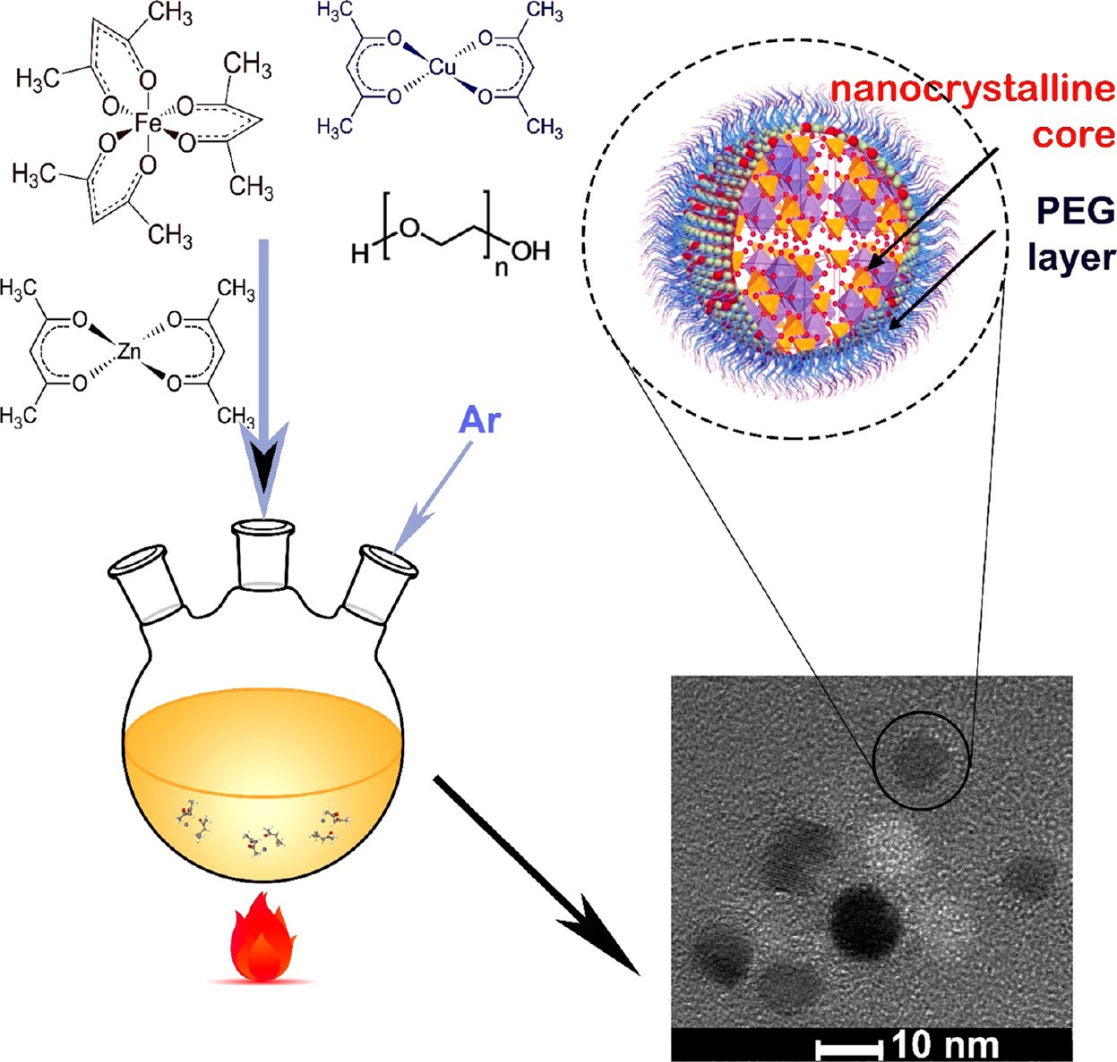
Schematic depiction of the one-step preparation of PEGylated nanoparticles. The left side illustrates the synthesis of Cu*_x_*Zn*_y_*Fe_3_*_x_*_–_*_y_*O_4_ ferrites. In one step, PEGylated nanoparticles are synthesized in a three-necked round bottom flask, where the thermal decomposition of organometallic precursors (Cu(acac)_2_, Zn(acac)_2_, Fe(acac)_3_) in a PEG polymer matrix (*M*_w_ = 1000 Da, 99%) under argon atmosphere takes place. The right side shows a cartoon of the nanoparticle’s structure and a TEM image of a few nanoparticles.

PEG surface-modified CuZn ferrite nanoparticles were obtained during a one-step synthesis based on the thermal decomposition of organometallic precursors (Cu(acac)_2_, Zn(acac)_2_, Fe(acac)_3_) in a PEG (*M*_w_ = 1000 Da, 99%) matrix under argon atmosphere, as graphically depicted in Figure 2. Three different water-stable copper–zinc ferrite nanoparticles were synthesized with intended three different Cu/Zn/Fe ratios (S1-0.4:0.6:2.0, S2-0.70:0.53:1.77, S3-1.15:0.40:1.45). In the first step of each synthesis, 7 mmol PEG was heated at 80 °C for 30 min under an argon atmosphere, stirring continuously on a magnetic stirrer. Appropriate amounts of Cu(acac)_2_, Zn(acac)_2_, and Fe(acac)_3_ precursors were then added to the molten PEG, in the molar ratio 1:7 (total amount of organometallic precursors to PEG1000). Each mixture was vigorously stirred at 80 °C under argon for 30 min. Then, the solution was quickly heated to 285 °C and kept at this temperature for 60 min. The obtained mixture was cooled to a temperature of 60 °C, and then 20 mL of toluene was added. After being cooled down to room temperature, the mixture was washed with acetone and diethyl ether. This mixture was purified by magnetic separation. The organic solvent was disposed of and replaced with pure water, in which nanoparticles were finally suspended.

### 2.14 Toxicology Studies

The murine fibroblasts (line NIH3T3) were cultured in an MEM high-glucose medium in a humidified incubator (37 °C and 5% CO_2_). The murine neuroblastoma cell lines were cultured in a Dulbecco’s modified Eagle’s medium (DMEM) high-glucose medium in a humidified incubator under standard conditions (37 °C and 5% CO_2_). The DMEM and MEM were supplemented with 10% fetal bovine serum (FBS) streptomycin (100 μg/mL) and penicillin (100 U/mL). The cells were subcultured every 2 days until the appropriate number of cells was obtained for testing. After the cells reached 80% confluence, they were trypsinized, seeded on sterile 96-well plates (4.0 × 10^4^ cells/cm^2^), and incubated for 24 h.

The cytotoxic activity of PEG-NPs in neuroblastoma and NIH3T3 cell lines was assessed by the 3-[4,5-dimethylthiazole-2-yl]-2,5-diphenyl tetrazolium bromide (MTT) dye conversion assay. Cells (4 × 10^4^) were cultured in a 0.1 mL volume of culture medium in a 96-well plate in the presence of different concentrations of PEG-NPs. After 24 h, the cells were washed once and further incubated for an hour with MTT dye. The obtained blue formazan precipitate was dissolved using a solubilization buffer (5 mM HCl in isopropanol) and kept for 2 h at 37 °C. The absorbance at 570 nm was measured using a microplate reader. Each result is presented as a mean, together with its standard deviation (SD), of the three independent experiments, each of them performed in triplicate. The significance of the differences between the cell viability values was determined with a Student’s t-test for two-group comparisons. In all of the cases, a probability value (*p*-value) of less than 0.05 was considered to be significant.

### 2.15 Data Processing

The NMR spectroscopic data, relaxation data, and MR images were processed using *Python*-based software developed in-house. A false-color temperature map was created using Origin (Origin 9.0, OriginLab Corporation, Northampton, MA). The statistical analysis of regression and correlation was conducted using the *Prizm* software (GraphPad Prism version 5.00 for Windows, GraphPad Software, San Diego, CA). The thermal simulation for the temperature gradient in the MRI setup was carried out using Energy2D Interactive Heat Transfer Simulation software (The Concord Consortium, Concord, MA).

## 3 Results and Discussion

### 3.1 Morphological and Chemical Characterization

Until now, most of the syntheses of PEGylated magnetic nanoparticles have been multistep syntheses. Typically, the first phase was the synthesis of nanocrystals, which was followed by the particle coating process. On the contrary, our process (for details, see the Experimental Section) allows us to stabilize the polymeric coating nanocrystals using a single-step method. A mixture of the precursor salts (Cu(acac)_2_, Zn(acac)_2_, and Fe(acac)_3_) with different ratios of metals and PEG (1000) was mixed. In this study, low-molecular-weight PEG1000 was used to avoid the accumulation of PEG in the liver and normal tissue lysosomes. Such accumulation was observed for high-molecular-weight PEG.^54^ The chemical composition of the obtained hybrid systems was investigated using the ICP-OES technique. The results are summarized in Table 1, which also shows the theoretical molar ratio of metals for each sample. The results of the ICP-OES measurements clearly show that the actual Cu content is much lower than expected. In the case of sample S1, only about 10–15% of the initial Cu atoms are incorporated into the spinel structure. It can also be seen that sample S1 contains less than 50% of the zinc that is incorporated into the ferrite structure. The low content of zinc and copper may be related to various reaction mechanisms that probably take place during the formation of nanoparticles. In this synthesis, copper(II) acetylacetonate, zinc(II) acetylacetonate, and iron(III) acetylacetonate were used without any other iron(II) source. Thus, the reaction of the reduction of Fe(III) to Fe(II) might disturb the copper and zinc incorporation in the spinel structure.^55^

**Table 1:** Molar Ratio of Various Metallic Components Measured by ICP-OES

	samples		
	S1	S2	S3
molar ratio	Cu/Zn/Fe	Cu/Zn/Fe	Cu/Zn/Fe
nominal	0.40: 0.60: 2.0	0.70: 0.53: 1.77	1.15: 0.40: 1.45
measured	0.08: 0.54: 2.38	0.36: 0.70: 1.94	1.22: 0.41: 1.37

**Figure 3:**
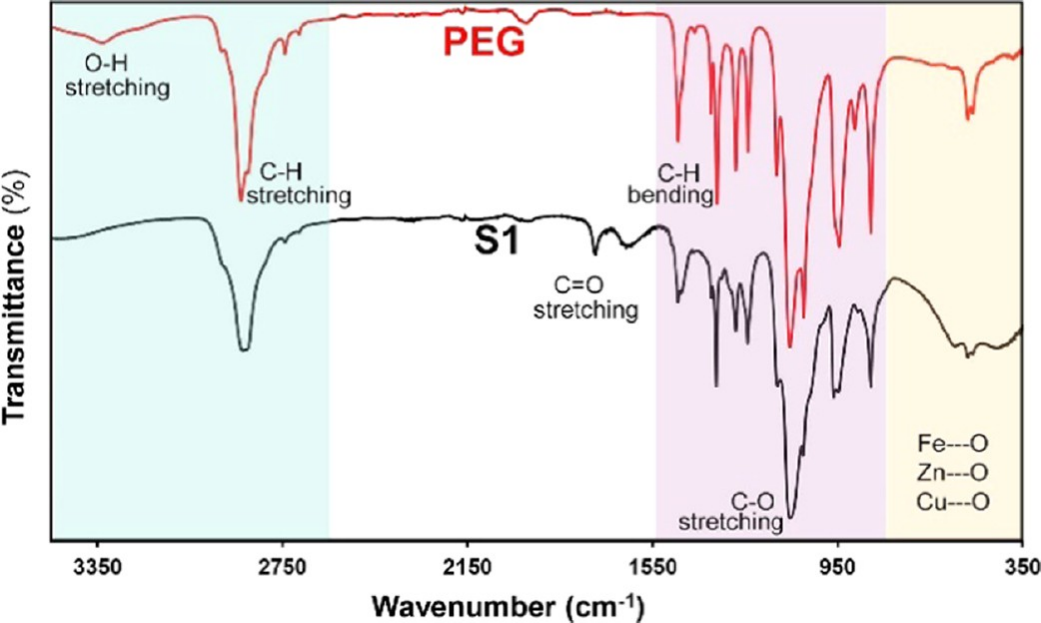
FTIR spectra of pure polymer PEG (red line), and S1 PEGylated nanoparticles (black line), recorded in the multiple internal reflection mode (i.e., as attenuated total reflection, ATR).

Fourier transform infrared (FTIR) spectroscopy and XPS measurements were performed to confirm the presence of the polymer coating on the surface of the nanoparticles after magnetic cleaning. The FTIR results confirmed the presence of PEG on the surface of the nanoparticles. The following peaks were observed on the spectrum of the unmodified polymer: peaks around 2957, 2888, and 2850 cm^–1^ are attributed to the alkyl chain of PEG1000; peaks at 1341 and 1108 cm^–1^ are due to C–H bending and C–O stretching vibration, respectively, while the peak at 1242 cm^–1^ corresponds to C–H twisting vibrations (Figure 3, red line).^52,53^ An example of the FTIR spectrum of the S1 system (nanoparticle core in a PEG matrix) is shown in Figure 3, where the presence of characteristic bands from PEG (such as the bands around 2891, 1344, and 1110 cm^–1^, corresponding to C–H stretching vibrations, C–H bending, and C–O stretching vibration) are clearly visible. All of these bands are shifted from their original position in unmodified PEG, which exhibits a hydrogen-bonding nature, confirming PEG’s interaction with the surface of CuZn ferrite nanoparticles. Moreover, absorption bands located at 525–540 cm^–1^ and in the region of 435–445 cm^–1^ were observed in the spectrum of nanoparticles (S1). These bands are associated with the presence of Fe–O bending vibrations in CuZn ferrites and with the tetrahedral and octahedral vibration of M^2+^/M^3+^ cations,^56^ respectively. Furthermore, the peak observed at 420 cm^–1^ corresponds to the octahedral stretching of copper ions Cu^2+^ (Cu–O), while the Zn–O bond is assigned to a stretching frequency at 544–554 cm^–1^.^56,57^

The samples used were in the form of powder. Different regions are highlighted to show the changes in the spectra associated with C–H stretching, C–H bending, and C–O stretching and vibrations associated with the Fe–O, Cu–O, and Zn–O bonds.

**Figure 4:**
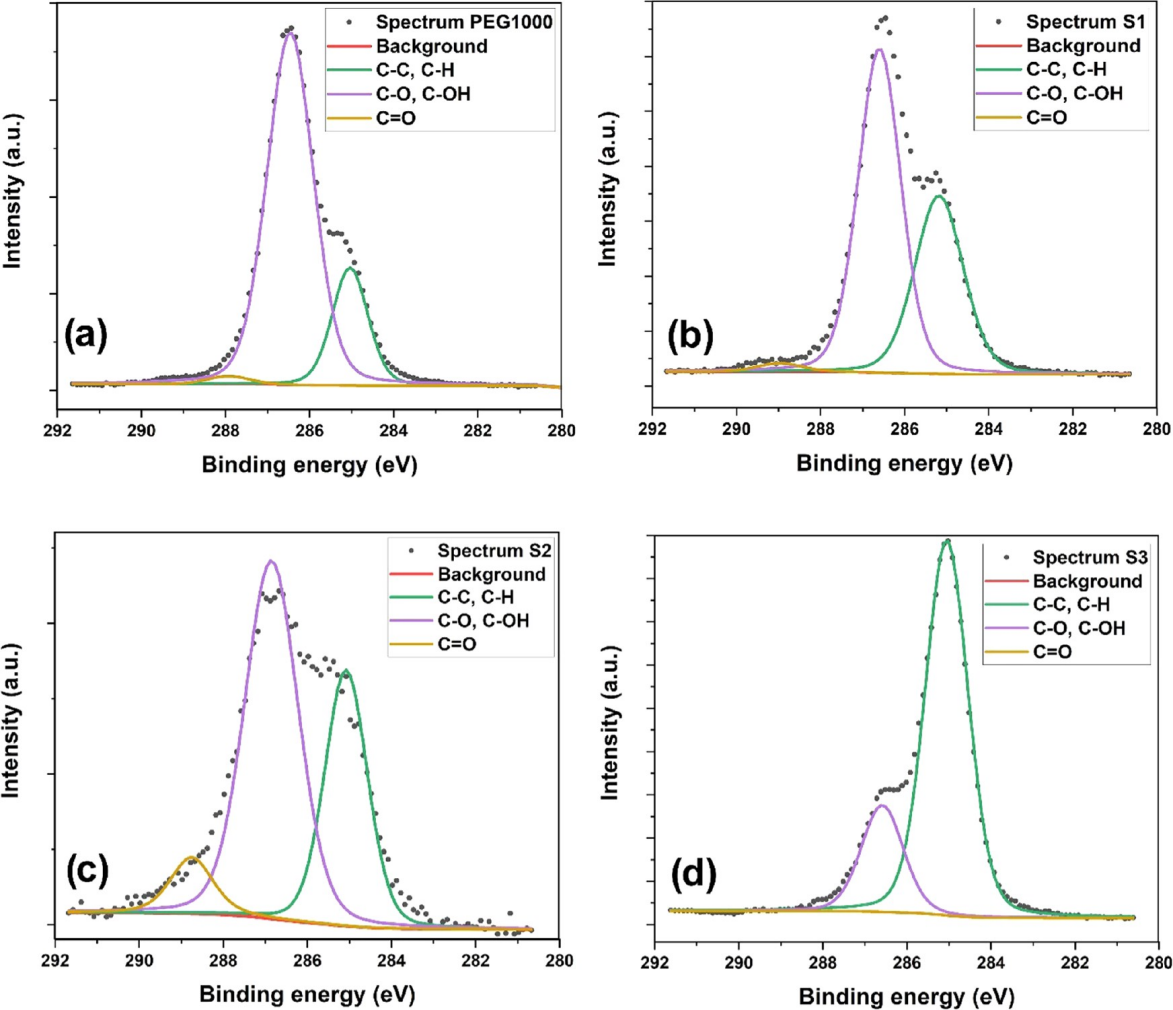
XPS spectra in the C 1s region for pure PEG1000 and PEG1000-coated nanoparticles. (a) Pure PEG1000, (b) S1 system, (c) S2 system, and (d) S3 system. The black symbols represent the measured C 1s spectra, while the red lines represent the background. The green, purple, and yellow solid lines show the fit components derived from the pristine material spectra, as indicated in the figures’ legends.

To further explore the structure of all PEGylated nanoparticle systems, high-resolution XPS spectra of the C 1s region were analyzed to determine the chemical states of carbon. Figure 4 presents high-resolution spectra measured for the pure PEG100 (Figure 4a), S1 (Figure 4b), S2 (Figure 4c), and S3 (Figure 4d) samples, respectively. All of the measured spectra were fitted with three peaks of 285.0, 286.6, and 288.6 eV binding energies. Figure 4a shows a C 1s spectrum measured for pure PEG1000 polymer. This data was fitted with two peaks with binding energies of 285 and 286.6 eV, attributed to the hydrocarbon C–C/C–H and ether carbon component C–O, respectively.^58^ The ether peak was significantly larger than the C–C hydrocarbon peak, which is related to the effect of attenuating photoelectrons emitted from the underlying carbon atoms.^59,60^Figure 4b presents the C 1s spectrum for the S1 sample, which shows that the 285.0 eV peak intensity increased. Furthermore, S1 nanoparticles show the presence of an additional peak in the C 1s spectrum at 288.6 eV, which can be associated with the O–C=O line. Moreover, in the S2 sample spectrum (see Figure 4c), the intensity of the O–C=O line (288.6 eV) and C–C/C–H line (285.0 eV) slightly increased, suggesting the degradation of ethylene oxide and the formation of functional groups corresponding to aldehyde and carbonyl carbon groups.

The obtained results confirm the earlier PEG studies, which showed that the degradation of the PEG chain leads to the reduction of the ether component in the C 1s region and the presence of a new carbonyl component at 288.6 eV.^61^ The scheme of the thermal degradation of PEG products, proposed by J.A. Hiltz,^62^ is visible in Figure 4c,d. Note that S3 nanoparticles show no presence of the O–C=O line (288.6 eV) in the C 1s spectrum. In addition, for sample S3, we observed a significant reduction in the intensity of the C–O peak and an increase in the intensity of the C–C peak. These results suggest that the increase in the content of copper ions in the studied ferrites contributes to a change in the structure of the polymer in the nanoparticle shell. It has been observed that for systems with increased copper content in the structure (S2 and S3), the polymer (PEG1000) degrades faster on the particle surface. PEG is a nonionic, polar, water-soluble polymer. We note that it has been widely used in many fields such as lubricants, pharmaceuticals, cosmetics, surfactants, and as a biodegradable reagent in metal extractions.^63^ Most literature ascribes the degradation of PEG to an oxidation mechanism, but some have concluded that this degradation is due to a thermal mechanism^64^ or even to high-speed stirring.^65^

**Figure 5:**
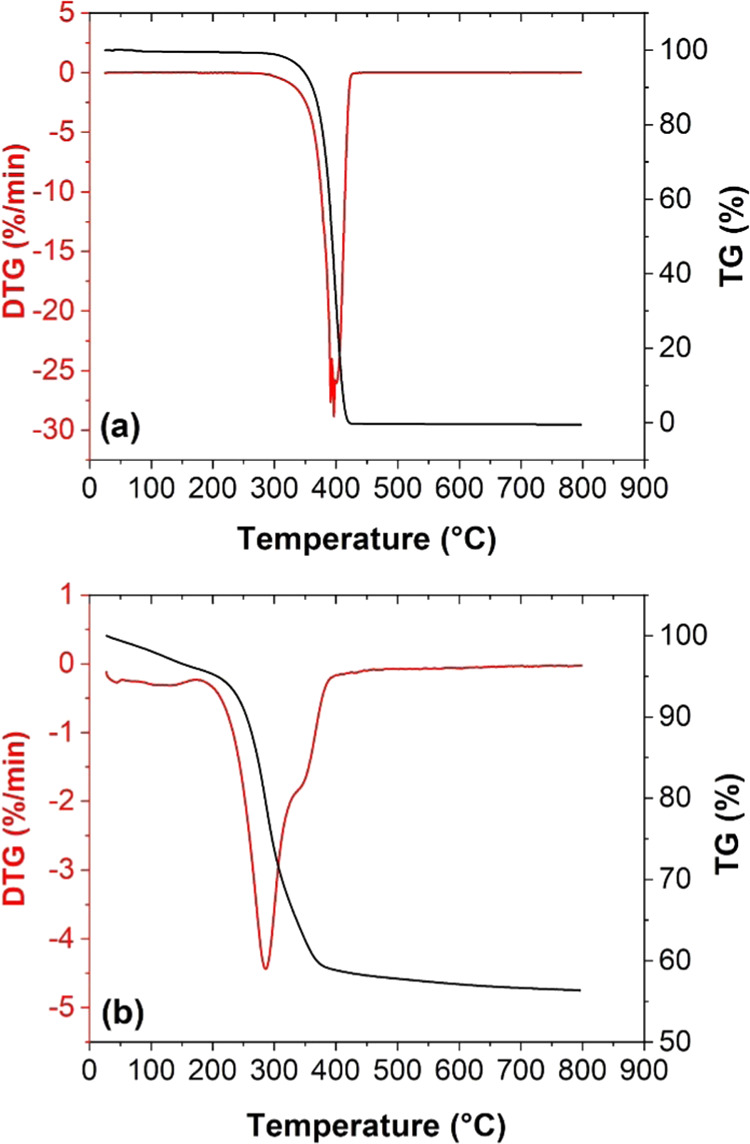
Thermogravimetric analysis of the selected materials. (a) Pure unmodified polymer PEG. (b) PEGylated nanoparticle type S1. The percentages (%) of the mass drops below and above 300 °C are given in the figures.

To better understand the interactions of organic medium (PEG) on the surface of nanoparticles, a thermogravimetry (TG)/differential thermogravimetry (DTG) analysis of all samples was carried out. The mass loss was measured while heating each powder sample up to 900 °C. Such measurements allowed to determine the amount of the organic compound surrounding nanoparticles. Figure 5 shows the results of the TGA analysis for the S2 sample carried out from room temperature to 800 °C. Characteristic inflections visible in the TG/DTG curves suggest the ongoing thermal decomposition of PEG that was adsorbed on the surface of the obtained nanoparticles. Evident weight decreases in the observed temperature ranges indicate that the temperature of the decomposition of PEG is about 300 °C. The largest weight loss was observed for the pure PEG1000 sample (>97%), whereas for sample S1, the weight loss was only 43.6%. This suggests that the surface of these nanoparticles is coated with the PEG polymer, confirming the formation of a PEG shell that is thermally degraded during the TG/DTG experiment. We note that the results of the TGA analysis were also used to properly determine the mass magnetization of CuZn ferrites covered with PEG.

**Figure 6:**
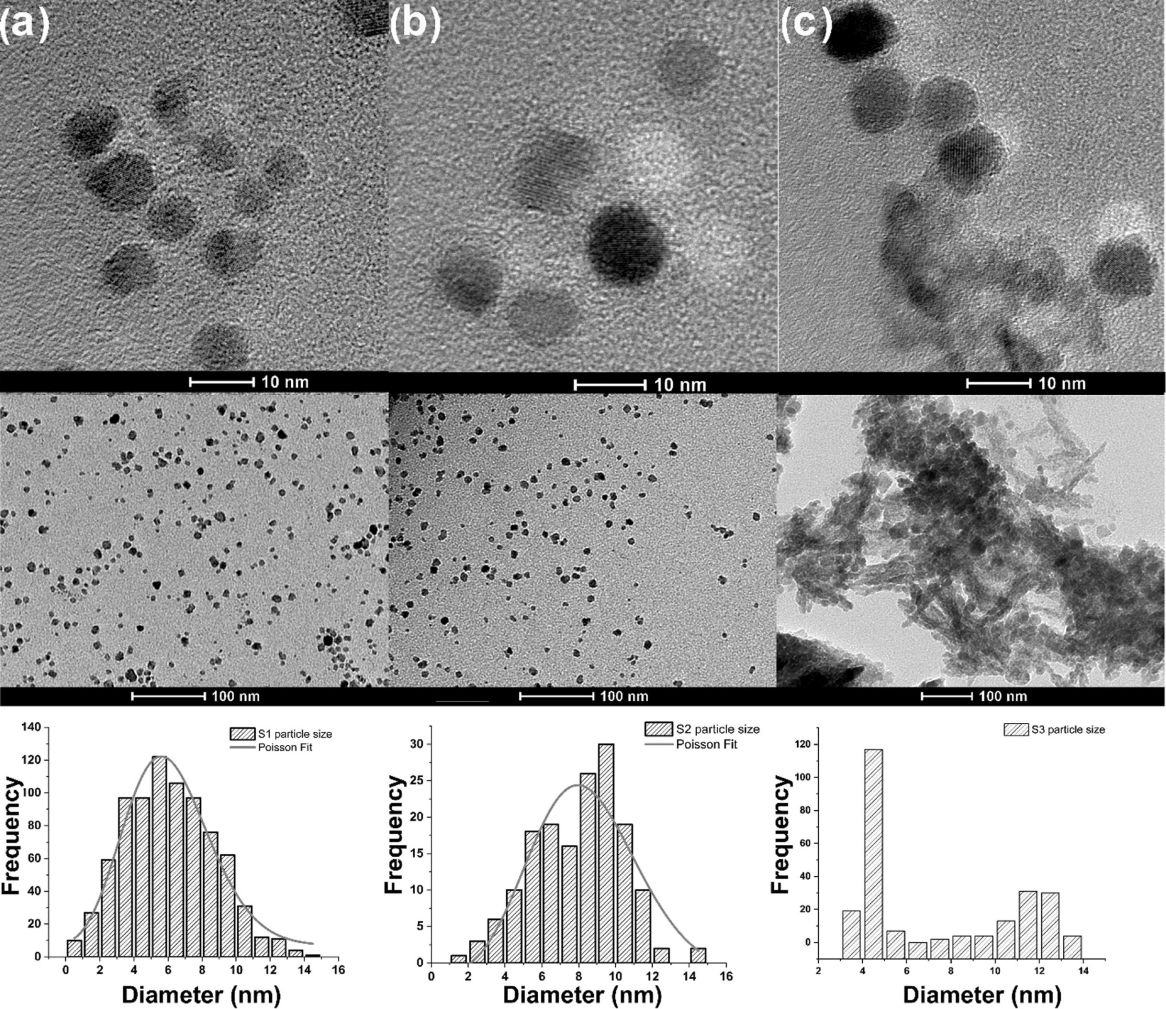
Imaging and analysis of the studied samples using TEM for the (a) S1 system, (b) S2 system, and (c) S3 system. Histograms of particle sizes have been generated and fitted to a Poisson size distribution for clarity.

To find the best composition or tMRI experiments, one needs to understand the morphology of the prepared CuZn ferrite nanoparticles coated with PEG. The panels in Figure 6 summarize the results from the TEM studies. Nanoparticles S1 and S2 were found to be morphologically finest among them (Figure 6a,b). For these particles, more uniform and less aggregated particles were observed. The smallest particle diameters, in the range of 3–9 nm, were recorded for the system S1. In the case of the S3 composition, with the highest copper content, an increased aggregation of particles was observed. In addition, a variety of particle shapes and increased polydispersity were observed in this system (Figure 6c). This effect may be related to the increased degradation of PEG in the polymer layer in this system.

**Figure 7:**
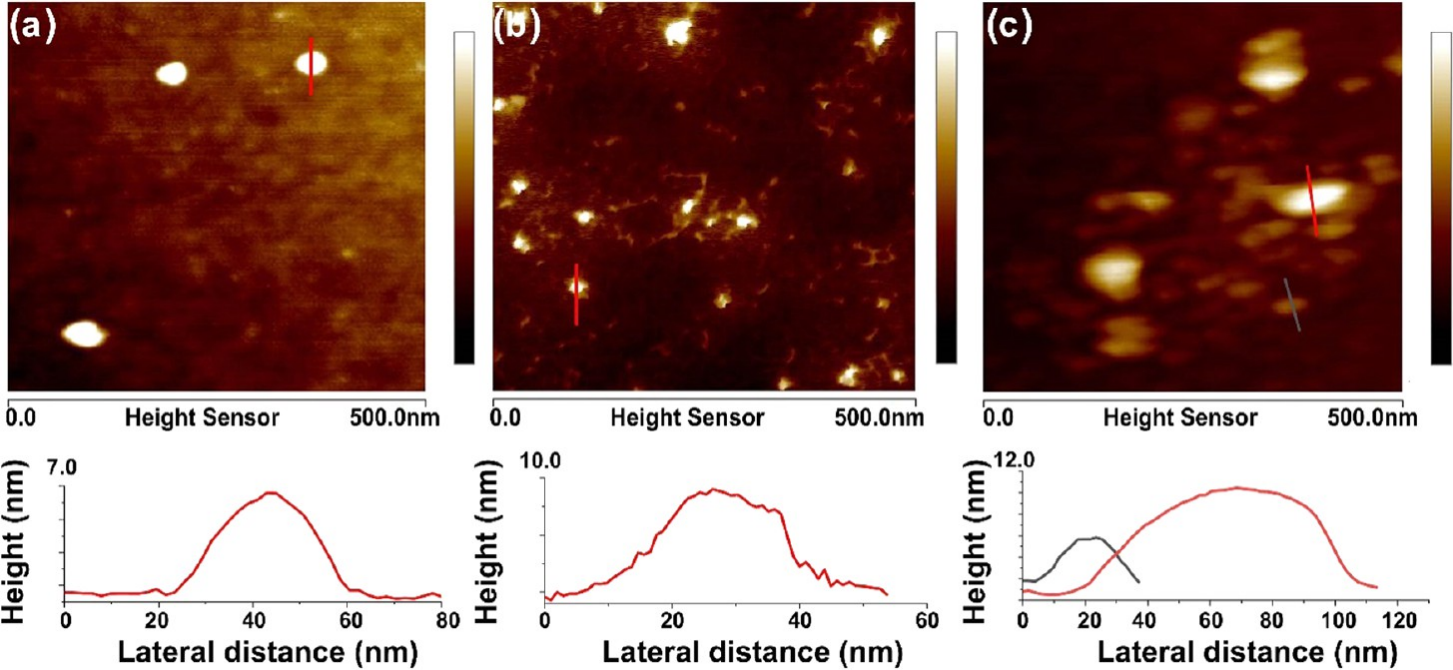
AFM images and examples of their intensity profiles of PEGylated nanoparticles. (a) S1 system. (b) S2 system. (c) S3 system.

The results of the atomic force microscopy measurements are presented in Figure 7. The analysis of the AFM images allowed defining the average size of the obtained particles with polymer shells. For sample S1, the AFM measurements revealed that the average size was around 20–35 nm, which is in good agreement with the DLS analysis (see Table 2). The AFM images of the S1 sample confirmed that spherical particles were obtained (see Figure 7a), which had a stable polymer surface (the AFM measurements did not damage the particle surface). The size (20–35 nm) of the obtained particles was slightly larger than that obtained from the TEM measurements, which was expected considering the presence of a surrounding polymer shell. The obtained cross-sectijoal profiles indicated a low surface roughness. For S2 particles (higher copper content), increased surface roughness and decreased adhesion of the shell to the substrate were found (as a result of the AFM measurement, the polymer was separated from the cores) (see Figure 7b). The AFM images of S3 (Figure 7c) presented aggregated particles of different sizes, which confirmed the results obtained in the TEM and DLS.

**Table 2:** Mean Hydrodynamic Diameter (*d_Z_*), Polydispersity Index (PDI), ζ-Potential (ζ) of Various PEG-MNPs Systems, Mean Diameter-Based TEM Data (*d*_TEM_), and Grafting Density of Nanoparticles Grafted with PEG (1000 g/mol) (σ_TGA_)

PEG-MNPs	*d_Z_* (nm)	PDI	ζ (mV)	*d*_TEM_ (nm)	σ_TGA_ (chain/nm^2^)
S1	21 ± 4	0.221	–28.6± 0.5	5.9 ± 0.1	4.49 ± 0.11
S2	16 ± 2	0.309	–11.7± 0.4	8.2 ± 0.3	3.83 ± 0.17
S3	419 ± 89	0.651	1.2± 0.6	12.0 ± 1.3	3.49 ± 0.32
				4.4 ± 0.5	1.28 ± 0.23

The hydrodynamic diameters of the obtained PEG-coated nanoparticles were tested using dynamic light scattering, while their ζ-potentials were measured with the same system using the electrophoretic light scattering (ELS) procedure. The results of ζ-potential measurements showed that the increase in copper content significantly influenced the stability of nanoparticles (see Table 2). The ζ-potential increased with increasing the copper concentration from −28.6 eV for S1 to +1.2 eV in S3. The DLS measurements for sample S3 have shown that the nanoparticles form aggregates in aqueous dispersion, and their hydrodynamic diameter was found to be equal to 419 ± 89 nm. The highest absolute value of the ζ-potential was found for the S1 system. The ζ-potential near −30 eV ensures higher colloidal stability of the aqueous dispersion. Therefore, we chose this S1 system for further biological and MRI studies.

The results of the morphological and structural characterization indicate that the one-step synthesis methods using only acetylacetonate and poly(ethylene glycol) (1000 Da) as precursors can provide a successful route for the production of nanoparticles. However, increased copper content in the reaction mixture might increase the thermal degradation of the poly(ethylene glycol) and prevent the effective stabilization of nanoparticles.

For all obtained systems, we calculated the grafting density of polymer chains on the nanoparticle (Table 2). The grafting density of PEG on nanoparticles (σ_TGA_) was calculated based on data from TGA analysis^66−68^ using eq 1.
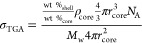
1The relative mass of the PEG (wt %_shell_) and the residual mass of the pure ferrite nanoparticles (wt %_core_) were determined at 800 °C. The number of polymer units per nanoparticle was obtained by taking the PEG mass and dividing it by the polymer mass per chain (i.e., PEG molecular weight/Avogadro’s number). The denominator in eq 1 requires a measure of the total particle surface area in the sample, a value defined here as a surface area per particle (4π*r*^2^) multiplied by the total number of particles. Nanoparticle number was obtained from the nanoparticle mass (wt %_core_) divided by the nanoparticle mass per particle, which corresponded to the product of the density of bulk ferrite (ρ_core_ = 5.0 g/cm^3^) and the volume of a single particle (4/3π*r*^3^).

All obtained nanoparticles systems were characterized in terms of size, surface charge, composition of the polymer layer, grafting density, and hydrodynamic volume, in particular, the effects of PEG grafting density and ζ-potential on colloidal stability. The results indicated that PEG grafting densities and the ζ-potential on the particles were different and depended on the core size and composition (copper content). The S1 system with the smallest core size, the degree of grafting of approx. 4.5 (chains/nm^2^), and the lowest ζ surface potential showed the greatest stability. The theoretical value for the maximum grafting density of the PEG chains is 4.54 (chains/nm^2^),^69^ assuming helical conformation PEG chains and its cross-sectional area of 22 Å^2^.^70^ However, for PEG-stabilized nanoparticle systems obtained by traditional two-step methods, usually lower values in the range of 0.5–1.5 are obtained.^71,72^ The high grafting density of PEG chains could be related to an improved synthetic method that allows the polymer chains to be densely packed by eliminating the solvent. The high grafting density of PEG chains may be related to an improved synthesis method that allows the polymer chains to be densely packed by eliminating the solvent.

### 3.2 Magnetic Properties

#### 3.2.1 Mössbauer and XAS Spectroscopy

**Figure 8:**
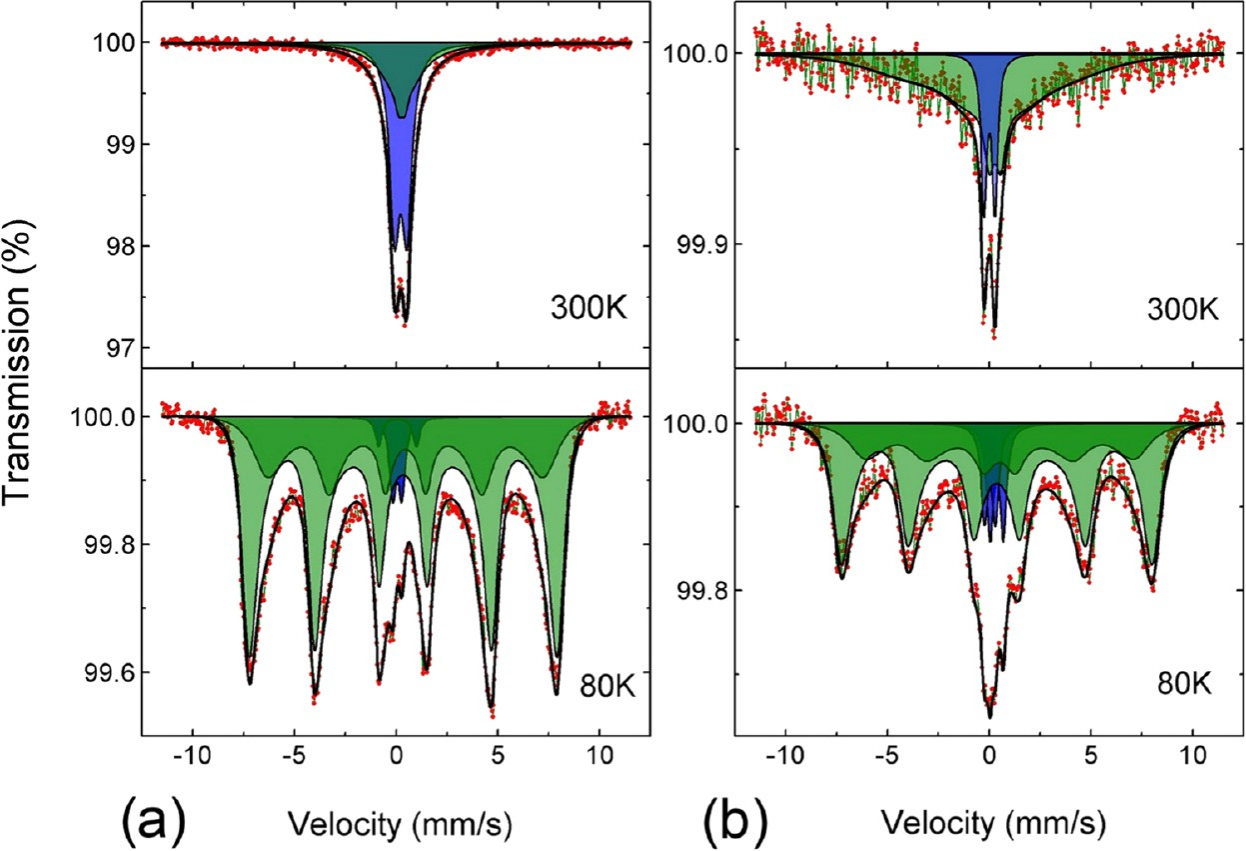
^57^Fe Mössbauer spectra measured at RT and 80 K. (a) S1 system and (b) S3 system. The red dots represent the experimental spectrum, while the black solid line is the numerical fit using individual components (shades of green and blue) corresponding to the contributions described in the text.

The Mössbauer spectra of the S1 and S3 samples, acquired at RT and 80 K, are shown in Figure 8. The spectra exhibit a relaxation character typical of superparamagnetic nanoparticles. The RT data were fitted with two components: (a) quadrupole doublet (blue) of a substantial contribution for S1 (69%) and a small contribution for S3 (20.5%), (b) Zeeman sextet (green) of relaxation character; a small contribution for S1, and large for S3, as well as an average magnetic hyperfine field (*B*_hf_) of 14.1, 28.1, and 38.0 kOe for S1, S2 (not shown in Figure 8), and S3, respectively. Both the large contribution of the magnetic component and the large average value of *B*_hf_ confirm that the nanoparticles of S3 are relatively large (slowly relaxing at RT), at least in comparison to S1 nanoparticles. The spectra measured at 80 K were fitted with four components: (a) two quadrupole doublets (blue components in Figure 8) of a small contribution (8.1% for S1 and 15.1% for S3 samples) and (b) two Zeeman sextets (green components) of relaxation character, large average *B*_hf_ (479 and 456 kOe) of a relative contribution 2:1, respectively, typical of such a ferrite. For the S1 sample, the average *B*_hf_ decreased from 468 kOe (at 80 K) to 141 kOe (at RT), whereas for the S3 sample, the reduction of *B*_hf_ is much smaller: from 462 kOe (at 80 K) to 380 kOe (at RT). Moreover, the contribution of the nonmagnetic component increased from 8.1% at 80 K to 69% at RT for the S1 sample, whereas for the S3 sample, the nonmagnetic component contributions are similar: 15.1 and 20.5% at 80 K and RT, respectively. The blue components correspond to the magnetization fluctuating faster than the characteristic time of Mossbauer measurement, which results in a magnetic hyperfine field averaged to zero. Since the fluctuation is thermally activated, the contribution of blue components increases with increasing temperature. The fitting results confirm that the relaxation is much faster for smaller S1 nanoparticles when compared with the relaxation observed for larger S3 nanoparticles.

The above interpretation agrees with the observed TEM images (and nanoparticle size distributions) shown in Figure 6. Moreover, the measured Mössbauer spectra are typical of ferrite compounds and the superparamagnetic properties of CuZn ferrite nanoparticles used for our experiments.

**Figure 9:**
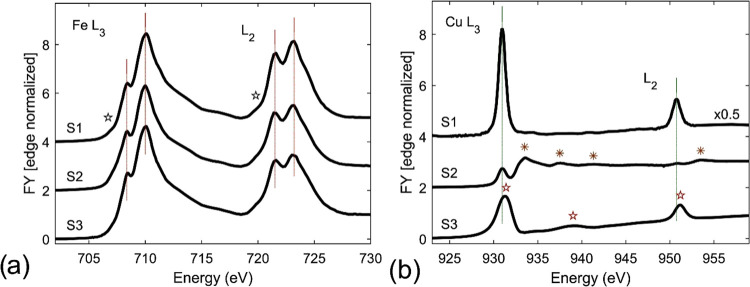
X-ray absorption spectra of the S1, S2, and S3 systems. (a) L edges of iron. (b) L edges of copper. Feature characteristics of Fe^3+^ and Cu^2+^ in the spinel oxide structure are marked with red and green dotted lines, respectively. Feature characteristics for metal ions with reduced oxidation, namely, Fe^2+^ and Cu^+^, are marked with the black and red pentagrams, respectively. Feature characteristics of metallic-like copper are marked with the stars.

An additional insight into the local atomic environment of Fe, Cu, and Zn ions, and thus the dominant structural phase in the composition of the synthesized particles, is obtained by X-ray absorption spectroscopy (XAS). XAS at the L edges of 3d metals is predominantly sensitive to crystal field symmetry and effective charge density (formal oxidation) averaged over all of the metal sites. Figure 9 shows the spectra probed at the Fe and Cu L edges using volume selective fluorescence yield detection. The Fe L-edge XAS of all of the studied samples is nearly identical. A complex spectral shape of both edges is a result of the convolution of the rich multiplet structure of Fe^3+^ ions in the octahedral and tetrahedral crystal fields.^73^ The double-peak structure at both edges with a tiny dip between the peaks at the L_3_ edge is typical of maghemite. It is further confirmed by the relative intensity of the peaks at the L_2_ edge. In addition, the spectra of all of the samples reveal tiny bumps in the pre-edge region of both edges (marked with black pentagrams in Figure 9), which are usually attributed to the presence of octahedral Fe^2+^.^74^ However, the relative intensity of these features with respect to the main resonances suggests that the fraction of Fe^2+^ ions, when compared with Fe^3+^ ions, is small (∼ 10%) in the case of the S1 sample and tiny in the case of the S2 and S3 samples. It is in agreement with the chemical stoichiometry determined using ICP-OES (see Table 1) for the S1 sample but rather unexpected in the case of S2 and S3 samples. This observation is further verified by ^57^Fe Mössbauer spectroscopy.

Contrary to Fe, the XAS spectra at the Cu L edge show significant shape differences among all of the studied samples. The spectrum of the S1 sample is a characteristic of Cu^2+^ ions occupying high inversion symmetry sites, as evidenced by a strong single peak structure at both edges (marked with green dotted lines in Figure 9b).^75^ Such a result is in line with copper embedded in the spinel oxide crystallographic structure. The spectrum of sample S2 shows some remaining of these features with additional structures at higher energies (marked with brown stars in Figure 9b). These features reveal a high resemblance to the spectrum of metallic copper. However, the relative intensity of the feature at ∼933 eV with respect to the other two is elevated, which might indicate the presence of Cu^+^ in an organic environment.^76^ The spectrum of the S3 sample at each edge consists of an asymmetric peak of lower intensity and the maximum at higher energy than that observed in the S1 sample. Moreover, a clear satellite peak is visible at an approximately 7 eV higher photon energy. The latter is characteristic of Cu in a molecular environment.^77^ It is most likely that Cu^+^ bound to products of the fragmentation of PEG.^78^ However, the asymmetry of the main feature might also be attributed to the small amount of Cu^2+^ in the spinel structure.

The results of the XAS investigations at the Fe, Cu, and Zn (data not shown) L edges imply the structure and chemical compositions of the studied nanoparticles as mixed CuZn ferrite. However, the relative fraction of Cu ions embedded in the ferrite phase (forming the core of nanoparticles) decreases from S1, through S2 to S3, which is contrary to the initially planned concentration. The results of the XAS analysis indicate the presence of a reduced amount of copper in samples S2 and S3. It may suggest the occurrence of a redox reaction between Cu^2+^ ions and aldehyde groups that come from the PEG degradation process. These data correlate well with the results from XPS, where the appearance of aldehyde groups in sample S2 and their disappearance in sample S3 was observed. In addition, for the S3 sample, we observed a significant reduction in the intensity of the C–O peak and an increase in the intensity of the C–C peak. This suggests the possible detachment of short fragments of the polymer chain from the nanoparticle and/or the formation of a pseudocorona ether complex with Cu^+^ ions, as postulated by Stoychev et al.^79^

#### 3.2.2 Magnetization Measurements

Based on the above-presented analysis of the studied samples, we determined that the nanoparticles of the Cu_0.08_Zn_0.38_Fe_2.54_O_4_ composition (S1) exhibit the best colloidal stability with the optimal particle diameter and homogeneous structure. We have thus chosen the S1 nanoparticles due to their small sizes and low polydispersity index (PDI) for further NMR and MRI investigations.

**Figure 10:**
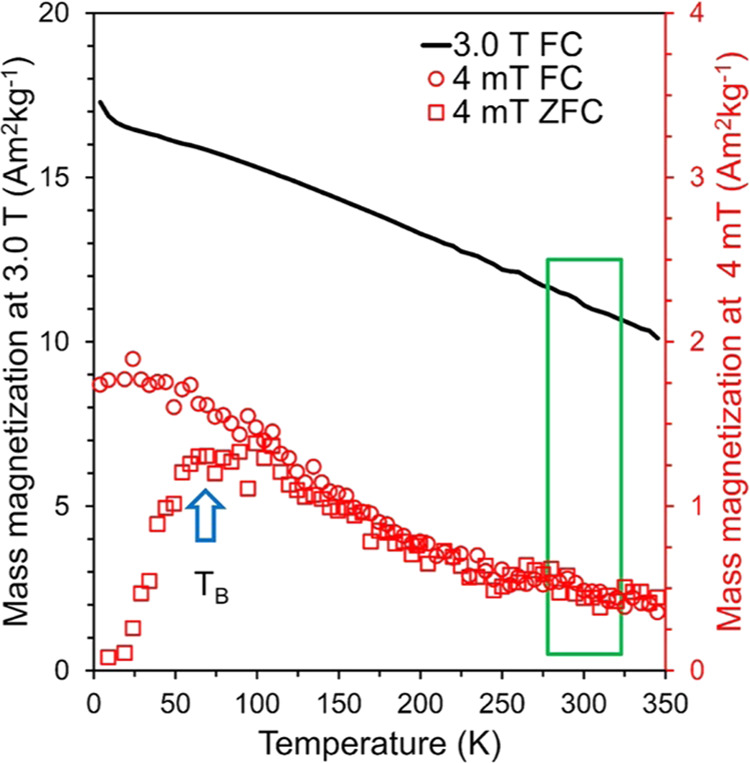
Temperature dependence of the mass magnetization of Cu_0.08_Zn_0.38_Fe_2.54_O_4_ at 3.0 T and at 4 mT with field cooling (FC) and zero-field cooling (ZFC) protocols. Note the magnetization maximum for ZFC, clearly defining the blocking temperature near 60 K. The green rectangle depicts the temperature region of interest for the NMR and MRI studies.

As mentioned above, the static magnetic properties were studied using a SQUID magnetometer. Figure 10 presents mass magnetization measurements as a function of temperature at the applied magnetic fields of 3.0 T and 4 mT. The low field (4 mT) measurements were carried out with field cooling and zero-field cooling protocols to observe the blocking temperature, T_B_.^80,81^ The S1 sample exhibits a superparamagnetic behavior with a blocking temperature near 60 K, visible as a maximum on a 4 mT zero-field-cooling data set. Such a low blocking temperature ensures that at temperatures above 300 K, even in a static applied field of 3.0 T, the magnetization of an individual particle fluctuates. Due to the dominance of thermal energy over monocrystalline energy, magnetization rapidly jumps among different easy directions.^82^ We note that these jumps of magnetization are sensed by protons of diffusing water molecules delivering a possible mechanism for their relaxation.^83^ The measurements at 3.0 T revealed a monotonic decrease of mass magnetization with temperature. The analysis of the correlation and linear regression shows a strong temperature correlation between mass magnetization and temperature (Pearson *r* = −0.9975, *p* < 0.01), with a high value of goodness-of-fit (*R*^2^ = 0.950) and a slope of −0.0222 ± 0.0004 Am^2^/kg/K.

#### 3.2.3 ^1^H Nuclear Relaxation

The mechanism for nuclear relaxation of water protons in aqueous gels with suspended superparamagnetic particles originates primarily from water molecules diffusion and particles’ magnetization fluctuations. The comprehensive theory of proton relaxation caused by superparamagnetic particles in aqueous suspensions and relevant experimental data are presented in the following selected refs (84−87).

**Figure 11:**
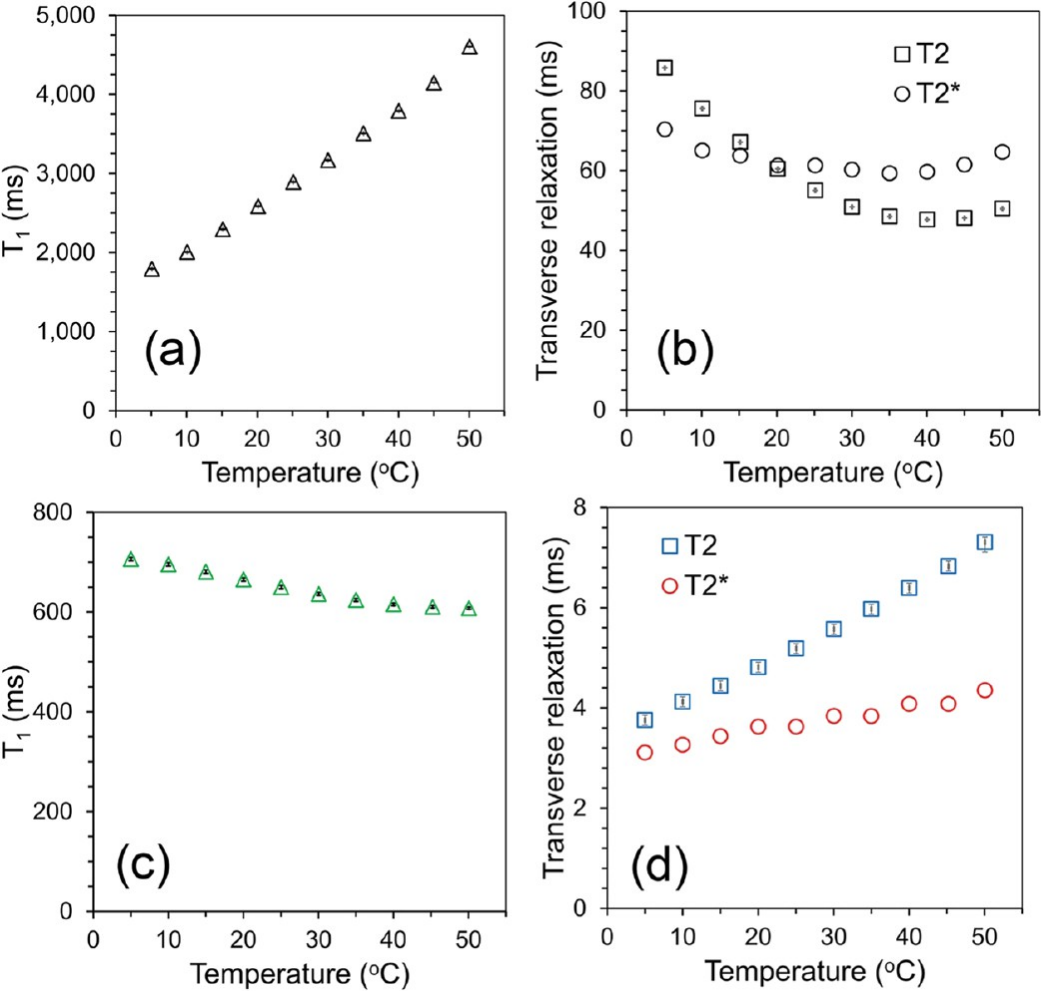
Temperature dependence of the water proton nuclear relaxation times *T*_1_, *T*_2_, and *T*_2_* at 3.0 T. (a,b) In pure 1% deionized water agar gel. (c,d) In agar gel with embedded S1 particles.

The results of the water proton nuclear relaxation measurements as a function of temperature in pure agar and agar doped with nanoparticles are shown in Figure 11. *T*_2_* was calculated from the observed NMR line width (υ_1/2_, full width at half-maximum) using the formula 2 ^88^
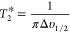
2Doping agar with nanoparticles only moderately changes the *T*_1_ values but completely reverses the thermal dependence with a nearly 15% decrease of *T*_1_ with increasing temperature. On the other hand, the presence of nanoparticles drastically changes the transverse relaxation in agar gel. Both the *T*_2_* and *T*_2_ values decrease approximately 10-fold with 0.85 mM doping. Moreover, *T*_2_ in doped agar shows a different temperature dependence than *T*_1_. There is nearly a 2-fold increase of *T*_2_ in the temperature range of 5–50 °C, while only a moderate increase of the *T*_2_* values (see Figure 11d).

We noted that the different temperature dependence in *T*_1_ and *T*_2_ of water protons in an agar gel with embedded particles provides a rare opportunity for the simultaneous use of *T*_1_ and *T*_2_ (or *T*_1_–*T*_2_*) weighting for the increase of MR image intensity changes with temperature. Such an effect resembles the proposed dual-mode *T*_1_–*T*_2_ contrast agents.^89−91^ However, unlike dual-mode agents designed to work only at one temperature of the human body, we are searching for nanoparticle compositions that will induce opposite and substantial wide-temperature changes of *T*_1_ and *T*_2_ in aqueous solutions, creating a sheerlike figure. More the *T*_1_–*T*_2_ “relaxation sheer” opens, the stronger is the temperature dependence of the MR image intensity, simultaneously weighted by *T*_1_ and *T*_2_. This effect can potentially lead to a much higher thermal and spatial resolution of the MRI thermometry method when magnetic nanoparticles with such properties are used.

#### 3.2.4 *T*_2_-Weighted Spin-Echo MRI

Because the temperature changes in the *T*_1_ values are small, we will primarily focus on the *T*_2_ increase with temperature as a possible mechanism to be employed for tMRI. We used a long repetition time of 4 s during MRI measurements, which is at least 5 times longer than the longest *T*_1_ time registered at 5 °C. Such a long repetition time prevents any *T*_1_ weighting (more than 99% of nuclear magnetization returns to the state of equilibrium along a static B_0_ magnetic field). We hypothesize that the increase in *T*_2_ time in the nanoparticle-doped agar gel will deliver changes in the image intensity due to *T*_2_ weighting in a spin-echo sequence.^85,92,93^ Since the *T*_2_ relaxation time is longer at higher temperatures, the observed voxels in warmer areas of the phantom should exhibit higher intensity (brightness), while the ones measured in colder parts of the sample should appear to be darker. Similar changes in the image intensity were observed in gel phantoms doped with micrometer size magnetic particles but using the *T*_2_*-weighted gradient-echo method that is inherently sensitive to local magnetic field inhomogeneity.^4,5,85^ Although traditional spin-echo sequences are generally slower than gradient-echo sequences, they are less prone to susceptibility and motion artifacts and facilitate excellent *T*_1_, *T*_2_, and proton density contrast in MRI.^92,94^ A major drawback of the spin-echo method is the long imaging time. However, this can be overcome by the acquisition of more than one echo after the excitation using a fast spin-echo sequence leading to imaging time shortening.^95^

**Figure 12:**
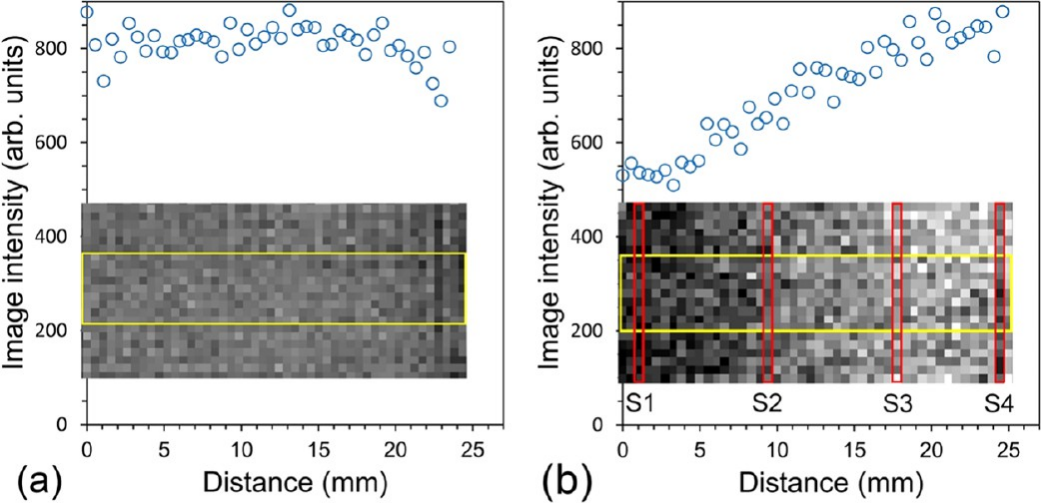
MR spin-echo image intensity (image intensity profile) as a function of position in the phantom. The image intensity is the average of the column of 9 pixels along the phantom within the yellow rectangle and projected to the corresponding column location in mm from the phantom bottom. The distance is measured from the phantom’s bottom. The images were rotated 90° clockwise to match the orientation of the profile. (a) At a uniform temperature of 18.1 ± 0.8 °C. (b) In the presence of the temperature gradient. The red bars show the positions of the temperature sensors. Note: the approximately 50% temperature increase in the image intensity for the doped agar is due to the temperature gradient.

We verified our hypothesis by imaging the agarose gel phantom doped with nanoparticles at a uniform temperature and in the presence of a strong temperature gradient using the setup described in the Microscopy Methods section. Figure 12 shows images of the same phantom in two different thermal conditions with the corresponding image intensity profiles above. The profiles were obtained by averaging the intensity from a region of interest (ROI) consisting of nine voxels selected from a certain location (column of 9 pixels) within the yellow rectangle and projected on the ROI distance from the bottom of the phantom.

The intensity of the MR images of a phantom at a uniform temperature of the magnet’s bore of 18.1 ± 0.8 °C shows a constant value of intensity, modulated by the noise, of 812 ± 37 in arbitrary units (Figure 12a). We note that there is no correlation between the image intensity and the ROI position: Pearson *r* = −0.2475 and two-tailed *P*-value = 0.11. Contrary to the uniform temperature case, in the strong temperature gradient, one can appreciate the increase in the intensity toward the warmer part of the phantom (see Figure 12b). The statistical analysis of the data acquired with the temperature gradient shows a strong correlation between the signal intensity at a given ROI and its position: Pearson *r* = 0.9644 and two-tailed *P-*value < 0.0001.

We provide a more quantitative analysis of the MRI experiment with the phantom in the temperature gradient below by associating the intensity of each voxel in the image matrix with temperature and presenting the final results in the form of a temperature map. As described in the Microscopy Methods section, the three spin-echo images were acquired during the phantom bottom-top cooling–warming cycle. Figure 12a shows the temperatures recorded by four sensors in the phantom during the imaging experiment. The initial oscillations in the temperature are random due to hands contacting the elements during the setup assembly (approximately 2 min).

To demonstrate that the temperature gradient in the phantom only changed in the vertical direction and not horizontally, we conducted additional on-the-bench measurements having the sensors in the horizontal plane in the center of the phantom. It was determined that the temperature changed by 1.04 °C laterally along 12 mm, with the temperature increasing from the center to the sides. The Teflon blocks remained located on the top and bottom of the phantom with similar initial temperatures as during the MRI experiment. This lateral gradient (0.09 °C/mm) was 10 times smaller than the vertical temperature gradient (1.11 °C/mm) and was neglected in the analysis of the image intensity discussed below.

**Figure 13:**
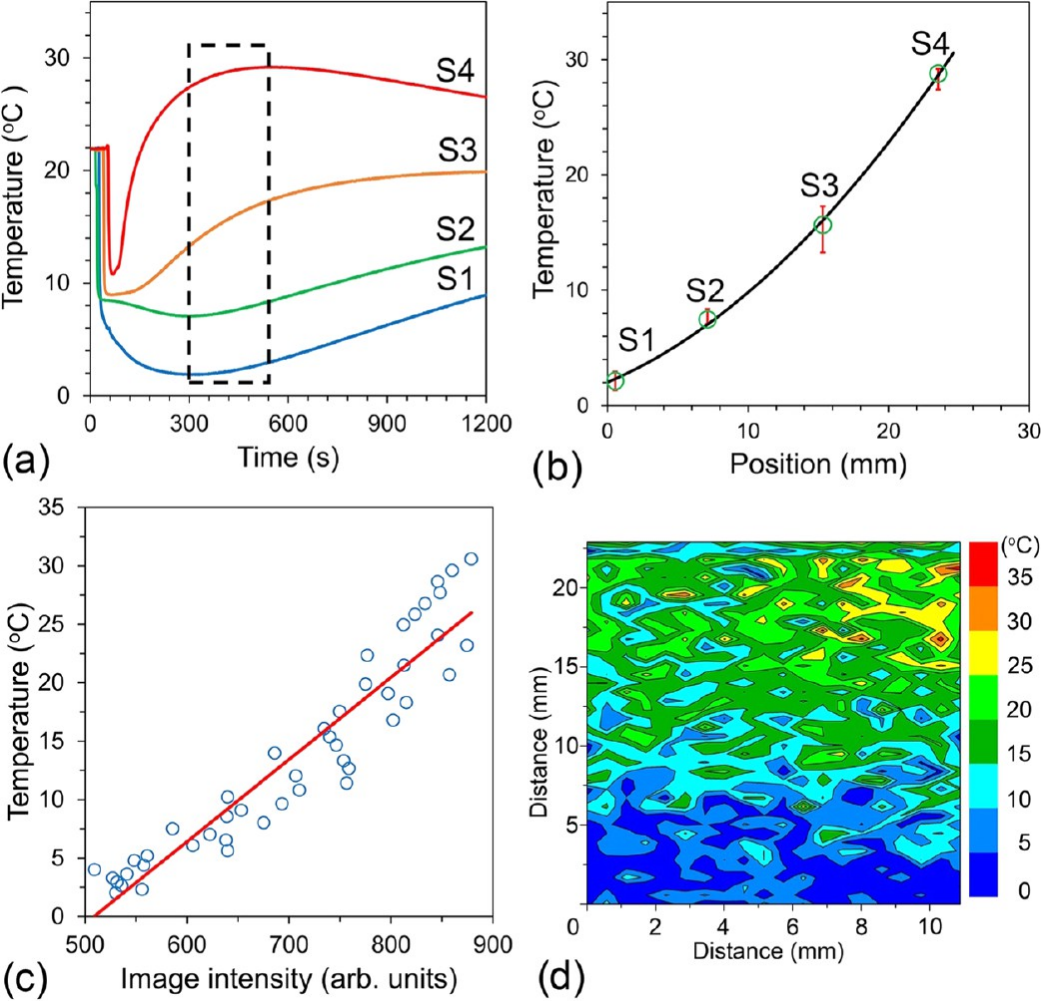
MRI in the presence of a strong temperature gradient. (a) Temperature changes in the phantom measured by sensors at four locations, as shown in Figure 1c. The dashed line rectangle delineates the time of the acquisition of the image used for the intensity analysis. (b) Quadratic polynomial fit to the four temperature values obtained from the sensors S1–S4 at the time corresponding to the middle of the image acquisition. (c) Temperature at the ROI as a function of the ROI intensity. The solid red line shows the linear fit. (d) Temperature map obtained by converting individual voxel intensity into temperature using the function *t* (°C) = 0.07*SI – 35.81, where the SI is the voxel intensity.

The initial simulations of the thermal behavior of the agar gel sandwiched between two Teflon blocks predict a quadratic temperature distribution with a temperature difference between the cold and hot spots reaching 30 °C (expected temperature gradient of 1.25 °C/mm). We used four experimental temperature (Figure 13a) values recorded at defined locations at the midtime of the MRI scan with the highest temperature gradient (the second scan of the three) and fit a quadratic polynomial to these values. The fit allowed us to obtain intermediate temperatures for the voxel locations through the length of the phantom (Figure 13b).

Next, we correlated the temperature with the image intensity of the results presented in Figure 13c as blue circles. We have found that such correlation is significant (Pearson *r* = 0.9427 and two-tailed *P* < 0.0001). The temperature versus intensity data from Figure 13c was then fitted to a linear function *t* (°C) = 0.07*SI – 35.81 (*R*^2^ = 0.88) to obtain an expression that was used to convert the signal intensity SI (arb. units) to temperature *t* (°C).

Using this expression and intensity data for the MRI uniform temperature case (812 ± 37 arb. units in Figure 12a), we calculated the temperature to be 21.0 ± 2.6 °C. The obtained value of temperature from the MRI intensity is in good agreement with the measured temperature of 18.1 ± 0.8 °C.

The same expression is used to convert the two-dimensional (2D) MRI signal intensity matrix to the color-coded temperature map. Figure 13d shows an example of such a conversion for the data presented in Figure 12a. The map shows a nonuniform pattern toward the phantom top. We note that there are two potential reasons for the observed nonuniformity. First, the phantom’s top did not have identical thermal contact over the entire surface with the heating block through an added layer of water. Second, the side of the phantom was warmed during the assembly of the phantom. We believe that the local temperature increase is real and further emphasizes the ability of the method to detect temperature changes.

### 3.3 Toxicology Studies

**Figure 14:**
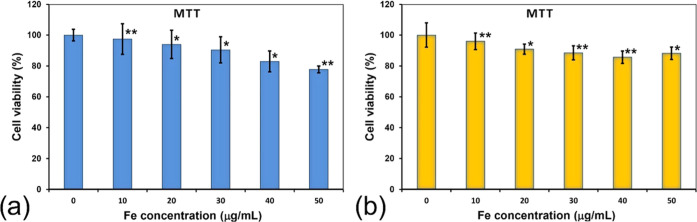
MTT assay test. (a) For NIH3T3, (b) neuroblastoma cells incubated for 24 h with nanoparticle S1. The cell viability is presented as a percentage of the control (cells incubated without nanoparticles). The error bars represent the mean and SD of the individual experiments performed in triplicate (*n* = 3). * *p* < 0.05; ** *p* < 0.01 compared with the control.

The studies of the cytotoxicity of PEG-coated nanoparticles (S1) were carried out on mouse fibroblasts (NIH3T3) and neuroblastoma cells. The viability of the cells was tested using the MTT assay. The fibroblasts were selected to represent a general toxicity screen. The results are shown in Figure 14. For both cell lines, no toxic effect was observed in the range of the concentrations tested. At the 50 μg/mL concentration, the nanoparticle system was relatively nontoxic as the viability remained in the 80–85% range. In the culture medium, the nanoparticles were stable, and their increased sedimentation was not observed, as in the previously reported studies.^6,96,97^ Also, no changes in the morphology of the stimulated cells were observed.

## 4 Conclusions

Our work demonstrates that water-stable CuZn ferrite superparamagnetic nanoparticles can be prepared using one-step thermal decomposition synthesis in poly(ethylene glycol). Our results revealed that the polymer shell structure on CuZn ferrite nanoparticles differs depending on the amount of copper used during the synthesis. The observed PEG shell degradation is caused by competing processes such as thermal degradation of PEG and the reduction of Cu^2+^ to Cu^+^ (with the formation of copper(I) complex compounds) or Cu^0^. It was also found that particles with a low content of copper Cu_0.08_Zn_0.54_Fe_2.38_O_4_ show long-term colloidal stability in water due to the effective coating of the nanoparticle core with nondegraded poly(ethylene glycol). The lack of significant toxicity of PEG-coated Cu_0.08_Zn_0.54_Fe_2.38_O_4_ MNPs was also confirmed by the experiments conducted on mouse neuroblastoma and NIH3T3 cell lines. The simplicity of the synthesis, excellent physical–chemical characteristics of the nanoparticles, and their auspicious magnetic and biological properties make PEG-coated ferrite particles a promising contrast agent for MRI thermometry.

The ferrite core component of the S1 system (Cu_0.08_Zn_0.54_Fe_2.38_O_4_) is nontoxic. The shell component (PEG) is stable and not biodegradable. After the use for contrasting, the intact particles will be exerted from the body.^98,99^ However, some PEGylated therapeutic agents induce the development of anti-PEG antibodies (APA), leading to reduced efficacy and severity of side effects.^100−102^ The immunogenicity of PEG and occasional induction of APA responses is poorly understood and is a subject of intensive research. Many factors, such as molecular size of PEG, linker type, or PEG grafting density on the nanoparticle surface, are implicated in the potential APA response.^103^ This could influence the final *in vivo* applications.

The preliminary MRI experiments using agar gel phantoms doped with Cu_0.08_Zn_0.54_Fe_2.38_O_4_ nanoparticles at a concentration of 0.85 mM and exposed to a temperature gradient of 1.1 °C/mm show significant changes in the intensity of *T*_2_-weighted spin-echo MR images. The spatial maps of phantoms with absolute temperature distribution can be obtained noninvasively from these changes in the MR image intensity. We estimate the uncertainty of the method to be 2.6 °C at 20 °C. Such a method is limited to known particles with known concentrations, and for practical use, it requires one temperature reference point and initial calibration. Since image intensity also depends on MRI sequence parameters, the calibration must be linked to specific sequence values such as the repetition time and echo time.^9,52,104,105^ In certain applications, such as clinical tMRI guided thermal ablations, the temperature measurement time is critical and needs to be in the range of a few seconds.^2^ We note that if our method is used in clinical settings, the well-defined human body temperature of 37 °C can be employed as a reference point. In this project, we mostly focused on proving the concept that ultrasmall, coated nanoparticles can be used as temperature contrast agents in MRI thermometry using a spin-echo sequence. Such issues as the acquisition time and temperature calibration will be addressed in future publications.
